# Amplification of Genomic DNA for Decoy Receptor 3 Predicts Post-Resection Disease Recurrence in Breast Cancer Patients

**DOI:** 10.14740/wjon764w

**Published:** 2014-03-11

**Authors:** Chizuko Kanbayashi, Yu Koyama, Hiroshi Ichikawa, Eiko Sakata, Miki Hasegawa, Chie Toshikawa, Naoko Manba, Mayuko Ikarashi, Takashi Kobayashi, Masahiro Minagawa, Shin-ichi Kosugi, Toshifumi Wakai

**Affiliations:** a Division of Digestive and General Surgery, Niigata University Graduate School of Medical and Dental Sciences, Niigata, Japan; b Department of Breast Oncology, Niigata Cancer Center Hospital, Niigata, Japan

**Keywords:** DcR3, Breast cancer, Fas-mediated apoptosis, Recurrence-free survival

## Abstract

**Background:**

Decoy receptor 3 (DcR3), a member of the tumor necrosis factor receptor (TNFR) superfamily, shows inhibitory effects on Fas-mediated apoptosis. Currently, data are lacking on the correlation between DcR3 and the recurrence of breast cancer. The authors examined DcR3 mRNA expression and genomic amplification in breast cancer, and investigated the effect of DcR3 gene amplification on prognosis of patients.

**Methods:**

A total of 95 patients formed the basis of the current retrospective study. DcR3 mRNA expression in breast cancer tissues was examined by RNase protection assay and *in situ* hybridization. DcR3 gene amplification was examined by quantitative polymerase chain reaction. The correlation between DcR3 gene amplification status and clinicopathological factors was examined and also the relationship between DcR3-Amp and relapse and survival.

**Results:**

The relative copy numbers of DcR3 genomic DNA correlated significantly with the levels of DcR3 mRNA expression (ρ = 0.755, P = 0.0067). In addition, lymphatic invasion correlated significantly with DcR3 gene amplification (P = 0.012). However, there was no correlation between the remaining clinicopathological factors and DcR3 gene amplification. In the univariate analysis, the recurrence-free survival (RFS) rate of patients who were positive for DcR3 gene amplification was significantly lower than that of patients who were negative for DcR3 gene amplification (P = 0.0271). Multivariate analysis showed that DcR3 gene amplification (P = 0.028) and disease stage (P < 0.001) remained significant independent predictors of RFS.

**Conclusions:**

DcR3 gene amplification was significantly correlated with lymphatic invasion, and also DcR3 gene amplification predicts recurrence after resection, which may be an important prognostic factor in breast cancer patients.

## Introduction

Humans have several defence mechanisms to prevent tumor progression, with the Fas/Fas ligand (Fas/FasL) system being one of the major and best-known mechanisms for evading tumor cells. The Fas/FasL system plays an important role in tumor cell death caused by cytotoxic lymphocytes [1]. However, tumors may grow and cause disease progression because the tumor cells counteract the host defence mechanisms. Decoy receptor 3 (DcR3), which shares the extracellular motif of the tumor necrosis factor receptor (TNFR) superfamily, inhibits Fas-mediated apoptosis. DcR3 gene amplification has been reported in lung and colon cancers [2]. The amplification and expression of DcR3 occurs in virus-associated lymphomas [3]. DcR3 overexpression has also been observed in human gastrointestinal tract cancer [4], glioma [5] and pancreatic cancer [6].

DcR3 overexpression may occur without genomic amplification [4], whereas DcR3 mRNA and protein overexpression appear to be positively related to gene amplification in astrocytic brain tumors, particularly glioblastomas [7]. DcR3 expression correlated with the grade of malignancy of 15/18 glioblastomas (WHO grade IV) but 0/11 diffuse astrocytomas (WHO grade II) [5]. However, few studies have looked at DcR3 expression or amplification in breast cancer patients. Therefore, we investigated DcR3 mRNA overexpression and gene amplification in breast cancer patients, and examined the relationship between DcR3 gene amplification and the clinicopathological factors of the patients. We also investigated the effects of DcR3 gene amplification on cancer recurrence and patient survival.

## Materials and Methods

### Patient population

The present study comprised 100 consecutive Japanese patients who underwent surgical resection for primary breast cancer during the 5-year period between January 1996 and December 2000. Four patients with distant metastases and one patient with inflammatory breast cancer who underwent neoadjuvant chemotherapy resulting in the absence of cancer cells in the resected specimens were excluded. The remaining 95 patients (94 women and 1 man) with median age of 56 years (range, 30 - 87 years) formed the basis of the current retrospective study. Consent was obtained from all individuals who participated in the study.

Surgical resection procedures depended mainly on the size of the primary tumor. We decided that patients with tumors larger than 3 cm should undergo modified radical mastectomy, while patients with tumors ≤ 3 cm but without wide intraductal spreading or multicentric lesions should receive breast-conserving surgery. Patients with DCIS, advanced age or poor general condition were not subjected to axillary lymph node dissection.

Sixty-five patients underwent modified radical mastectomy, 22 patients received breast-conserving surgery, three patients underwent mastectomy without axillary dissection, three patients underwent wide resection without axillary dissection and two patients underwent radical mastectomy.

In all, 72 patients received some form of adjuvant therapy after resection: 50 patients received hormone therapy (tamoxifen, LH-RH agonist plus tamoxifen), nine patients received chemotherapy (CMF and CAF) and 13 patients received chemohormonal therapy. Only one patient received neoadjuvant chemotherapy (CAF).

### Patient follow-up after resection

After surgical resection, the patients were followed up every 3 - 6 months in outpatient clinics and were monitored for disease recurrence by means of physical examinations and laboratory tests. The patients underwent routine imaging (mammography and/or breast ultrasonography (US), chest X-rays, abdominal US/CT and bone scintigraphy) every 12 months. When tumor recurrences were detected, the patients were administered either hormone therapy (for example tamoxifen, aromatase inhibitors, MPA) or chemotherapy (for example AC, docetaxel, paclitaxel). In some cases of brain and/or bone metastasis, radiation therapies, including gamma-knife, were also introduced. For some of the patients with locoregional recurrence in the breast or axilla, local resection was performed.

The follow-up period after surgical resection was defined as the interval between the date of initial treatment and that of the last follow-up, which ranged from 4 to 105 months (median, 70 months) in the current series.

### PCR-based cloning

The cDNA fragments for human Fas (360 bp; nt +137 to +496), human FasL (377 bp; nt +256 to +632), human DcR3 (389 bp; nt +386 to +774) and human glyceraldehyde-3-phosphate dehydrogenase (115 bp) were derived from human oesophageal cancer cells by RT-PCR cloning and inserted into the pGEM 3Z vector (Promega Corp., Madison, WI, USA). The recombinant plasmids were linearized with the appropriate restriction enzymes and used as templates for *in vitro* transcription of [^32^P]- or [^35^S]-labeled antisense cRNA probes, which were used in the RNase protection assay (RPA) or *in situ* hybridization (ISH) experiments, respectively.

### RPA

The RPA was performed to detect and semi-quantitate the levels of DcR3, Fas and FasL mRNAs in the breast cancer tissues, as reported previously [8]. In brief, total RNA (10 µg) from each breast cancer sample was hybridized with [^32^P]-labeled Fas, FasL or DcR3 probe combined with the GAPDH probe at the specific radioactivity level of 1 × 10^4^ cpm, overnight at 48 °C. Then, unhybridized probes were digested with ribonuclease A and ribonuclease T1 at 30 °C for 1 h, and the ribonucleases were digested with proteinase K at 37 °C for 30 min. After phenol/chloroform extraction, the hybridized probes were precipitated with ethanol, denatured at 85 °C and electrophoresed in 6% polyacrylamide gels. The dried gels were exposed to X-ray films for 3 days. The density of each band was analyzed by densitometry using the NIH Image software (version 1.59), and the expression of DcR3 mRNA was defined as the ratio of DcR3 mRNA to GAPDH mRNA band density, which was greater than 3% in the present study.

### ISH

ISH was performed to determine the distribution of DcR3 mRNA in breast cancer tissues, as reported previously [9]. In brief, cryostat sections (10 mm thickness) of human breast cancer specimens were fixed with 4% paraformaldehyde in PBS, treated with 3 mg/mL proteinase K solution for 10 min at room temperature and hybridized with [^35^S]-labeled cRNA probes (1 × 10^6^ cpm/section) overnight at 55 °C. After washing with buffer at room temperature, the sections were treated with 20 mg/mL RNase A solution for 30 min at room temperature, followed by washing with buffer at 55 °C for 4 h and at room temperature. After dehydration, the sections were exposed to a photographic emulsion for 5 - 7 days in the dark at 4 °C, developed, counterstained with hematoxylin and observed by both bright- and dark-field microscopy.

### Quantitative PCR

Quantitative PCR was used to measure DcR3 gene amplification, as reported previously [4]. In brief, genomic DNA samples from breast tissues (95 cancer tissues and 12 non-cancerous tissues) were isolated from paraffin-embedded sections by microdissection under a light microscopy and using the DNA isolator PS kit (Wako Pure Chemical Industries, Osaka, Japan). Quantitative PCR was carried out using the ABI PRISM 7700 sequence detection system (Applied Biosystems, Foster City, CA, USA). The DcR3-specific primers were 5'-CTTCTTCGCGCACGCTG-3' and 5'-ATCACGCCGGCACCAG-3', and the fluorogenic probe was 5'-ACACGATGCGTGCTCCAAGCAGAA-3' [2]. The β-globin-specific primers were 5'-ACCCTTAGGCTGCTGGTGG-3' and 5'-GGAGTGGACGATCCCCAAA-3', and the fluorogenic (internal control) probe was 5'-CTACCCTTGGACCCAGAGGTTCTTTGAGTC-3'.

For DcR3 genomic DNA quantitation, the amplification reactions (25 mL) contained isolated genomic DNA (0.5 µL), 1 × TaqMan universal PCR master mix (Applied Biosystems), 900 nM DcR3 primer and 250 nM of the corresponding fluorogenic probe. For β-globin genomic DNA quantitation, the amplification reaction (25 µL) contained isolated genomic DNA (0.5 µL), 1 × TaqMan universal PCR master mix (Applied Biosystems), 900 nM β-globin primer and 250 nM of the corresponding fluorogenic probe. Two-step PCR was performed according to the manufacturer’s instructions under the following thermal cycler conditions: 50 °C for 2 min, 95 °C for 10 min, followed by 40 cycles of 95 °C for 15 s and 60 °C for 1 min. The colon adenocarcinoma cell line SW480 [4] was run on each PCR plate as a positive control. No-template controls were also run on each PCR plate.

The data were analyzed using the SDS software (Applied Biosystems) and are presented as the means of the DcR3 amplicon/β-globin amplicon ratios.

### Prognostic factors

In addition to DcR3 gene amplification, we included several clinicopathological factors in the analysis of prognosis. The clinicopathological factors employed in the present study were: age (≤ 50 years versus > 50 years), pT, pN, pStage, lymphatic invasion, venous invasion, estrogen receptor (ER), PgR, Her2 and adjuvant therapy. Information on these factors was obtained from the medical records of each patient. The causes of death were determined from the medical records, and deaths from other causes were treated as censored cases. The follow-up period was defined as the interval between the date of treatment and the last follow-up. The correlation between DcR3 gene amplification and other clinicopathological factors was also examined.

### Statistical analysis

Medical records and survival data were obtained for all 95 patients. Survival curves were constructed using the Kaplan-Meier method, and differences in survival were evaluated using the log-rank test. The Cox proportional hazards regression model was used to identify factors that were independently associated with RFS. In this model, a stepwise selection is used for variable selection, with entry and removal limits of P < 0.05 and P > 0.01, respectively. The stability of this model was confirmed using step-backward and step-forward fitting procedures; the variables identified as having an independent influence on survival were identical for both procedures. The clinical features and pathological tumor-related factors were compared between the two patient groups using Fisher’s exact test. All statistical evaluations were performed using the SPSS 12.0J software package (SPSS Japan Inc., Tokyo, Japan). All tests were two-sided, and differences with P values of < 0.05 were considered statistically significant.

## Results

### Measurements of DcR3, Fas and FasL mRNA expression levels by RPA

DcR3 mRNA was prominently expressed in 50% (4/8) of the cases, as assessed by RPA. Fas mRNA was expressed in all of the cases, in which DcR3 mRNA expression was detected by RPA. In contrast, FasL mRNA was not detected in all of the cases, in which DcR3 mRNA expression was detected by RPA (data not shown).

### DcR3 mRNA distributions measured by ISH

**Figure 1:**
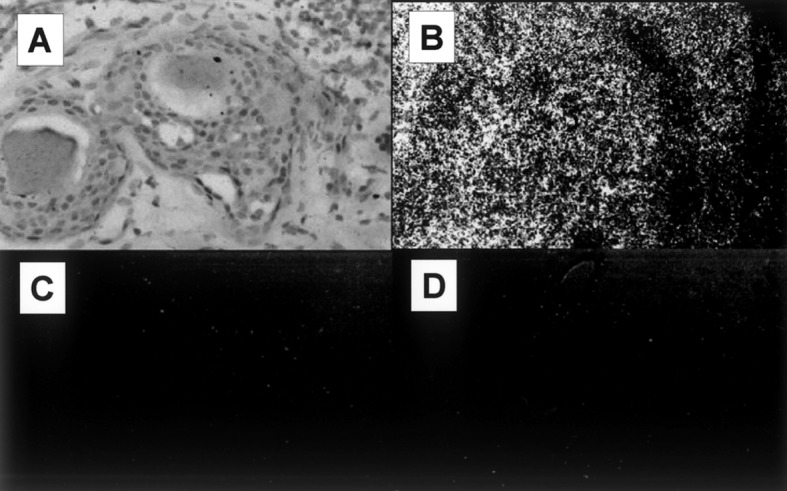
DcR3 mRNA expression analysis by ISH. (A) Light microscopy of breast cancer cells. (B) DcR3 mRNA expression is prominent in breast cancer cells, as visualized with the antisense probe. (C) The signals for the sense probe are negligible in the breast cancer cells. (D) The signals for the antisense probe are negligible in the normal breast cells.

In the ISH using the antisense probe, DcR3 mRNA expression was found to be prominent in breast cancer cells. The signals were negligible in breast cancer cells and normal breast tissues tested with the sense probe or antisense probe. These results show that DcR3 mRNA is detectable in breast cancer cells by ISH (Fig. 1).

### Relationship between DcR3 gene copy number and DcR3 mRNA expression

**Figure 2:**
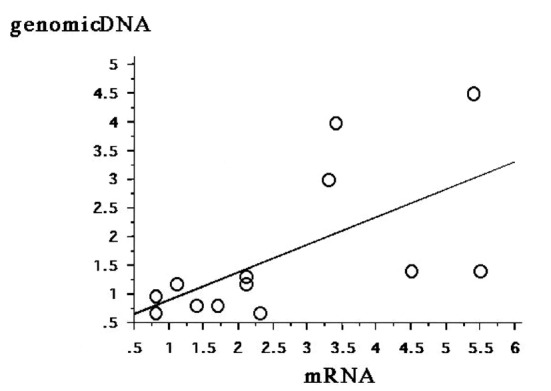
Correlation between DcR3 gene amplification and DcR3 mRNA expression. The relative copy number of DcR3 genomic DNA correlates significantly with the level of DcR3 mRNA expression (ρ = 0.755, P = 0.0067).

DcR3 mRNA expression was extensive in five cases and faint in nine cases, as assessed by RPA (data not shown). The five strong cases had higher relative copy numbers of DcR3 genomic DNA than the nine faint cases (data not shown). The relative copy number of DcR3 genomic DNA correlated significantly (ρ = 0.755, P = 0.0067) with DcR3 mRNA expression (Fig. 2).

### DcR3 genomic amplification in cancerous and non-cancerous breast tissues

**Figure 3:**
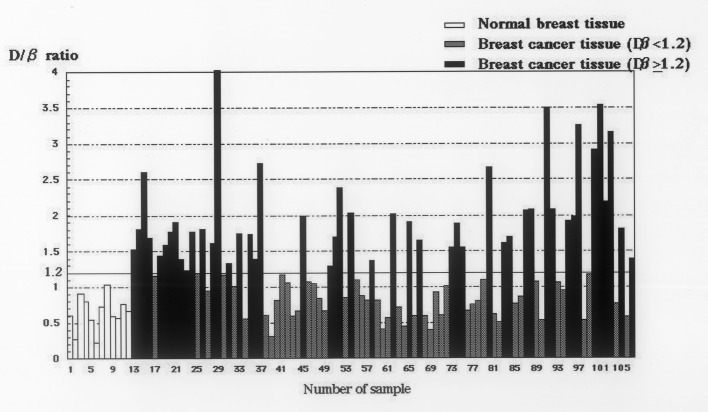
Relative copy numbers of DcR3 genomic DNA in 95 breast cancer tissues and 12 non-cancerous tissues. The median relative copy number in 95 breast cancer tissues is 1.19. DcR3 gene amplification was defined as ≥ 1.2 DcR3 copies in this study. The median relative copy number in 12 non-cancer tissues is 0.64.

The median relative copy number of DcR3 in the 95 breast cancer tissues was 1.19. Therefore, in the present study, positive DcR3 gene amplification was defined as ≥ 1.2 DcR3 copies, whereas negative DcR3 gene amplification was defined as < 1.2 DcR3 copies. The median relative copy number of DcR3 in the 12 non-cancer tissues was 0.64 (Fig. 3).

### Association between DcR3 gene amplification and other clinicopathological factors

The patients were subdivided into two groups according to DcR3 gene amplification status: 1) positive for DcR3 gene amplification (n = 47); and 2) negative for DcR3 gene amplification (n = 48). The clinicopathological characteristics were comparable for the two groups, with the exception that lymphatic invasion was observed more frequently (P = 0.012) in patients with positive DcR3 gene amplification (Table 1).

**Table 1:** Association Between DcR3 Gene Amplification and Clinicopathological Factors

Variable	No. of patients		P value
	Positive for DcR3 genomic DNA	Negative for DcR3 genomic DNA	
Age (years)			0.561
≤ 50	14	18	
> 50	33	30	
pT stage			0.532
pTis-T1	27	31	
pT2-T4	20	17	
pN stage			0.089
pN0	26	35	
pN1-pN3	21	13	
pTNM-stage			0.208
pStage 0-II	35	41	
pStage III	12	7	
Lymphatic invasion			0.012
Negative	32	43	
Positive	15	5	
Venous invasion			0.232
Negative	39	44	
Positive	8	3	
ER			0.152
Negative	26	19	
Positive	21	29	
PgR			0.062
Negative	32	23	
Positive	15	25	
Her2			0.767
Negative	42	41	
Positive	5	7	
Adjuvant therapy			0.339
No	9	14	
Yes	38	34	

DcR3: decoy receptor 3; ER: estrogen receptor; PgR: progesterone receptor; Her2: c-erbB2 receptor.

### Factors influencing RFS after surgical resection

**Figure 4:**
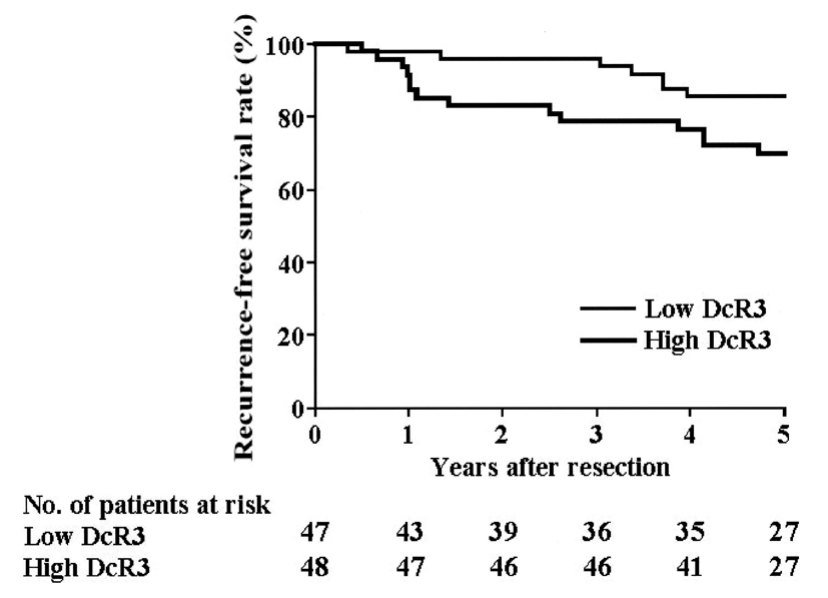
Recurrence-free survival. The RFS of patients with DcR3 gene amplification is significantly lower than that of patients lacking DcR3 gene amplification, as assessed in the univariate analysis (P = 0.0271).

The cumulative RFS rate was 77.5% at 5 years post-resection. Univariate analyses revealed that stage (P < 0.0001), nodal status (P = 0.0018), ER status (P = 0.0269) and positive DcR3 gene amplification (P = 0.0271) (Fig. 4) were statistically significant prognostic factors for RFS (Table 2). All 11 variables were entered into a multivariate analysis, which revealed that positive DcR3 gene amplification (P = 0.028) and pStage (P < 0.001) remained as significant independent predictors of RFS (Table 2).

**Table 2:** Univariate and Multivariate Analyses of Clinicopathological Factors and DcR3 Gene Amplification With Respect to RFS

Variable	No. of patients	5-year RFS (%)	Univariate analysis	Multivariate analysis	
			P value	Relative risk (95% CI)	P value
DcR3 genomic DNA			0.0271		
Low	48	85		1.00	
High	47	70		2.898 (1.121 - 7.487)	0.028
ER			0.0269		
Negative	45	68			
Positive	50	86			
pN			0.0018		
pN0	61	87			
pN1-3	34	61			
pStage			< 0.0001		
pStage 0-II	76	86		1.00	
pStage III	19	45		8.638 (3.244 - 23.003)	< 0.001

DcR3: decoy receptor 3; RFS: recurrence-free survival; CI: confidence interval.

### Factors influencing survival after surgical resection

The cumulative overall survival (OS) rate was 84.5% at 5 years post-resection. Univariate analyses revealed that stage (P = 0.0005), nodal status (P = 0.0030), venous invasion (P = 0.0111), ER status (P = 0.0155) and lymphatic invasion (P = 0.0206) were statistically significant prognostic factors for survival. All 11 variables were entered into multivariate analyses, which revealed that pStage III (relative risk (RR): 3.270; 95% confidence interval (CI): 1.162 - 9.200; P = 0.025; and ER-positive status (RR: 3.349; 95% CI: 1.061 - 10.577; P = 0.039) remained as significant independent predictors of survival.

### Correlation between DcR3 gene amplification and patient prognosis

Twelve patients died of tumor relapse, four patients died of other causes (for example hepatocellular carcinoma, cardiac infarction, rectal cancer and mycosis fungoides), eight patients were alive with recurrent disease and the remaining 71 patients were alive without recurrence.

## Discussion

Although DcR3 overexpression and gene amplification have been reported previously, the correlation between DcR3 expression and prognosis remains controversial. In gastric cancer patients, DcR3 overexpression is associated with significantly shortened OS [10], whereas there is no correlation between DcR3 expression and disease-specific survival in urothelial cancer patients [11].

There are very few reports of DcR3 expression in breast cancer patients; a single report concluded that DcR3 mRNA overexpression does not occur in breast tumors [4]. However, in the present study, we show by RPA that DcR3 mRNA is expressed in breast cancer tissues, and we reveal by ISH the distribution of DcR3 mRNA in cancer cells. DcR3 mRNA was expressed in half (4/8) of the cases, and DcR3 mRNA was detected by ISH in breast cancer cells. We assume that the lower number of tested breast cancer specimens accounts for the negative findings for DcR3 expression reported previously.

We also examined the relationship between the DcR3 gene copy number and DcR3 mRNA expression, since it is easier to obtain genomic DNA from the paraffin-embedded samples of previously treated patients than to obtain mRNA, and analysis of prognosis is possible using the previous samples of patients, for which the clinical course is already apparent. Our results show that the relative copy number of DcR3 genomic DNA significantly correlates with DcR3 mRNA expression. Since we demonstrated a significant correlation between DcR3 gene amplification and DcR3 mRNA overexpression, we could introduce DcR3 gene amplification into further studies of the analysis of prognosis for breast cancer patients.

Of the clinicopathological factors examined, DcR3 gene amplification showed a significant correlation only with lymphatic invasion. Since DcR3 has been shown to have an inhibitory effect on Fas-mediated apoptosis [2] and the Fas/FasL system is introduced by cytotoxic lymphocytes [1], our present results on the relationship between DcR3 status and lymphatic invasion appear to be appropriate and plausible. However, in the present study, no significant relationship was observed between DcR3 status and nodal status. Currently, it is difficult to explain why DcR3 status correlates with lymphatic invasion but not with nodal status.

In the present study, we also examined the effects of several clinicopathological factors on the RFS and OS of the breast cancer patients. Several clinicopathological factors are known to be important in the prognosis of breast cancer patients, including nodal status [12, 13], tumor size [14-16], histological and nuclear grades [17-20], histological subtype [21-23], ER status [24, 25], Her-2/neu expression [26, 27] and peritumoral vascular invasion [28-31]. For the relationships between these factors and DcR3, the univariate analysis shows that not only ER status, nodal status and stage, but also DcR3 gene amplification are significant prognostic factors of RFS in breast cancer. Furthermore, our multivariate analysis reveals that DcR3 gene amplification is an independent prognostic factor with significant effect on RFS. However, we could not prove the significance of DcR3 gene amplification on OS in this series of breast cancer patients. Although ER status, lymphatic invasion, vessel invasion, nodal status and stage correlated significantly with OS in the univariate analysis, DcR3 gene amplification did not correlate with OS in the univariate and multivariate analyses. It is difficult to explain the observed discrepancy regarding the effects of DcR3 gene amplification on RFS and OS in this series of patients. A possible explanation is that the population size of 95 patients or observation time of 5 years is not sufficient to detect a significant effect of DcR3 gene amplification on OS. Our result of P value of univariate analysis of DcR3 on OS was, with larger patients number and/or prolonged observation time, DcR3 may have been a significant prognostic factor on OS in breast cancer patients near future. This is the first study to demonstrate that DcR3 gene amplification is predictive of disease recurrence after resection in breast cancer patients.

### Conclusion

Our study has shown that amplification of the genomic DNA for DcR3 may be an important prognostic factor in breast cancer.
